# The inhibition of tyrosine kinase receptor signalling in leiomyosarcoma cells using the small molecule kinase inhibitor PTK787/ZK222584 (Vatalanib^®^)

**DOI:** 10.3892/ijo.2014.2683

**Published:** 2014-09-29

**Authors:** ANDREAS K.A. GAUMANN, HANNES C.A. DREXLER, SVEN A. LANG, OLIVER STOELTZING, SIMONE DIERMEIER-DAUCHER, ELISABETH BUCHDUNGER, JEANETTE WOOD, GUIDO BOLD, GEORG BREIER

**Affiliations:** 1 Institute of Pathology, Kaufbeuren-Ravensburg, 87600 Kaufbeuren, Germany; 2 Department of Molecular Cell Biology, Max Planck Institute for Physiological and Clinical Research, 61231 Bad Nauheim, Germany; 3 Institute of Virology and Immunobiology, University of Würzburg, 97070 Würzburg, Germany; 4 Department of Surgery, University Medical Center Regensburg, 93053 Regensburg, Germany; 5 Department of Obstetrics and Gynecology, University Medical Center Regensburg, 93053 Regensburg, Germany; 6 Novartis Oncology, CH-4002 Basel, Switzerland; 7 Institute of Pathology, Technical University of Dresden, 01307 Dresden, Germany

**Keywords:** leiomyosarcoma, receptor tyrosine kinase, small molecule inhibitor, PTK787/ZK222584, tumor, angiogenesis, VEGF, VEGF receptor

## Abstract

Leiomyosarcomas remain challenging tumors to manage and novel therapy strategies besides radiation and conventional chemotherapy are needed. Targeting angiogenesis by inhibition of vascular endothelial growth factor (VEGF) receptor tyrosine kinases (RTKs) of the tumor vasculature with small molecules is a promising new therapy. It has been shown recently that these receptors are not only expressed on tumor endothelium but also on tumor cells themselves. Thus, we investigated the expression of members of the VEGF receptor (VEGFR) family and corresponding growth factors in leiomyosarcoma tissue specimens and in the leiomyosarcoma cell lines SK-LMS-1 and SK-UT-1. We evaluated the influence of the VEGFR inhibitor PTK787/ZK222584 (PTK787) on cell growth, migration, apoptosis and phosphorylation of intracellular signalling molecules. In human leiomyosarcoma tissue specimens VEGFR-1/-2 and platelet-derived growth factor receptor (PDGFR-β) were strongly expressed. Both leiomyosarcoma cell lines expressed VEGFR-1/-3 and PDGFR-β but VEGFR-2 protein expression was positive only in SK-UT-1. SK-LMS-1 and SK-UT-1 cells secreted high and low amounts of VEGF-A, respectively, whereas PDGF-BB secretion was similar in both cell lines. Application of PTK787 led to partial inhibition of PDGF-BB-activated AKT/p90RSK and ERK1/2 signalling pathways. In contrast, protein phosphorylation was not affected by PTK787 in VEGF-A-treated cells. PTK787 turned out to inhibit cell migration even though no effects were observed upon stimulation with VEGF-A or PDGF-BB. In line, cell growth in leiomyosarcoma cell lines remained unchanged upon PTK787 treatment alone and with subsequent VEGF-A- or PDGF-BB-stimulation. However, VEGF-A, but not PDGF-BB-treated cells showed increased cell death upon PTK787 treatment. VEGFR family members are expressed in leiomyosarcomas *in vivo* and *in vitro*. Upon receptor stimulation, PTK787 is able to inhibit subsequent phosphorylation events and influences cell survival but not metabolic activity and migration. Thus, the inhibitor is possibly an additional option in the treatment of leiomyosarcomas.

## Introduction

Leiomyosarcoma is a highly malignant neoplasm that shows a high rate of local recurrence and distant metastasis, associated with aggressive growth and poor prognosis (1). The standard multimodal treatment strategies are surgery, radiation and conventional chemotherapy. Due to the insufficient effectiveness of the current treatment options, novel therapy options for treatment with target-specific drugs are urgently needed (2,3).

Inhibition of tumor-caused angiogenesis has emerged as a new therapeutic tool for therapy of diverse cancers. Growth of new blood vessels via release of angiogenic factors such as vascular endothelial growth factors (VEGFs) has been shown to be essential for tumor growth, nutrient supply and migration in metastasis (4). VEGFs signal through their cognate receptor tyrosine kinases (RTKs) (VEGFR-1/Flt-1, VEGFR-2/Flk-1/KDR and VEGFR-3/Flt-4). Importantly, it has been shown that VEGF receptor (VEGFR) family members are expressed not only in cells of the tumor cell microenvironment (vascular, lymphatic, endothelial and non-endothelial cells) (5) but on various cancer cells such as multiple myeloma, leukaemia, breast, colon, pancreatic (5–8) and leiomyosarcoma cells (2). VEGF-A has been demonstrated to have a stimulatory effect on prolife ration and migration of diverse VEGF-A-expressing carcinoma cells *in vitro* and *in vivo* (6,7,9–13). Thus, anti-angiogenic drugs for inhibition of angiogenesis and tumor cell growth are considered as promising alternative or supportive tools to conventional tumor therapy (4). Different strategies are available to repress tumor angiogenesis and have been approved for clinical use in diverse cancers, e.g., ligand-specific antibodies (bevacizumab) (14) or small molecule inhibitors (e.g., pazopanib, sorafenib, sunitinib) (4,15).

Inhibition of VEGFR family members has been proven to be effective in several malignancies (16–18). In particular, simultaneous inhibition of multiple, related RTK families was suggested to be a more efficient strategy for antitumor treatment compared to single receptor targeting (19). The multi-targeted tyrosine kinase inhibitor PTK787/ZK222584 (PTK787) (Vatalanib) has been shown to inhibit not only VEGFR-1, -2 and -3 but also platelet-derived growth factor receptor (PDGFR)-α and -β kinase activity (20). VEGF-induced phosphorylation of VEGFR-1, -2 and -3 *in vitro* is specifically blocked by PTK787, which leads to inhibition of endothelial cell proliferation, differentiation, tumor cell migration and VEGF- and platelet-derived growth factor (PDGF)-induced angiogenesis (6,20–25). Additional activity of PTK787 *in vivo (*26) has led to clinical trials in different malignant diseases. In a phase II and III trial, PTK787 treatment showed promising results in relapsed or progressing non-small cell lung cancer (27) and in a subgroup of metastatic colorectal cancer patients, respectively (14,18,28).

PTK787 exerts an antitumor activity on the tumor endothelium via reduction of vessel density in tumor tissues of many different entities (6,24). However, in order to understand the mechanism of action of the drug it is essential not only to focus studies on the effects of PTK787 on the tumor cell environment/vasculature but also on the tumor cells themselves, which were also shown to express VEGFR family members (2). In this study we evaluated the rationale for using the VEGFR tyrosine kinase inhibitor PTK787 in leiomyosarcoma cells. We found high expression of VEGFR family members and PDGFR-β in leiomyosarcoma tissue specimens and in the leiomyosarcoma cell lines SK-LMS-1 and SK-UT-1 in addition to ligand secretion. Intracellular signalling pathways were partially inhibited by PTK787. Leiomyosarcoma cell growth remained unchanged upon PTK787 treatment alone or in combination with VEGF-A or PDGF-BB. However, PTK787 treatment affected cell migration and cell death.

The expression of angiogenic growth factors, their corresponding receptors and functional responsiveness to inhibition of VEGFR/PDGFR signalling provides strong evidence that leiomyosarcoma patients with VEGFR- and/or PDGFR-positive tumor samples might benefit from anti-angiogenic treatment by inhibition of both autocrine stimulation of tumor cell growth and paracrine stimulation of angiogenesis.

## Materials and methods

### Cell cultures and reagents

Human umbilical cord vein endothelial cells (HUVECs) were isolated from human umbilical chords with a standardized protocol as described (29). Human leiomyosarcoma cell lines SK-UT-1 and SK-LMS-1, and human promyelocytic leukemia cells (HL-60) were obtained from the American Type Culture Collection (ATCC) (Manassas, VA, USA). Leiomyosarcoma cell lines were cultured under standard conditions in Dulbecco’s modified Eagle’s medium (DMEM) with high glucose content (PAA Laboratories GmbH, Pasching, Austria) and supplemented with 10% fetal calf serum (FCS) (CCPro, Oberdorla, Germany), 2 mM glutamine and penicillin/streptomycin (both from PAA Laboratories GmbH, Cölbe, Germany). HUVEC cells were isolated and cultured under standard conditions in MCD131 medium as previously described (29,30). The tyrosine kinase inhibitor PTK787 was provided by Novartis AG (Dr J. Wood, Oncology Research Group, Basel, Switzerland) and was developed as a joint venture of Novartis AG and Schering AG (Berlin, Germany). A 100 mM stock solution was prepared in DMSO and stored at −20°C. For all assays the inhibitor was diluted in culture medium to a final concentration as indicated. The concentration of DMSO was diluted to 0.1% for all assays. Recombinant VEGF165 (cat. no. 300-076; ReliaTech GmbH, Braunschweig, Germany) and recombinant PDGF-BB (cat. no. GF018; Chemicon International, Temecula, CA, USA) were applied at final concentrations of 100 or 50 ng/ml, respectively.

### Analysis of mRNA expression with RT-PCR

VEGFR-1, -2 and -3, and PDGFR-β mRNA expression was assessed in leiomyosarcoma cell lines with RT-PCR. Briefly, RNA was extracted using the RNeasy mini kit (Qiagen, Hilden, Germany) following the manufacturer’s instructions. RNA concentration was quantified by UV spectrophotometry. RT reaction was performed with Superscript^®^ Reverse Transcriptase (Invitrogen Life Technologies, Darmstadt, Germany) according to the manufacturer’s protocol. Subsequently, cDNA was amplified in a RoboCycler^®^ (Stratagene, San Francisco, CA, USA) using sequence specific primers (Table I) (Eurofins MWG Operon, Ebersberg, Germany) and Taq polymerase (cat. no. M1245; Promega GmbH, Mannheim, Germany) with a precycle of 4 min at 94°C and an amplification reaction of 35 cycles (94°C for 1 min, 58°C for 1 min and 72°C for 2 min). The reaction was terminated by 7 min at 72°C. Expression of GAPDH was used as a control to measure the integrity of the RNA samples. To exclude DNA contamination, purified RNA was incubated with the appropriate primers and Taq polymerase, but without reverse transcriptase. cDNA isolated from HUVEC and HL-60 cells was used as positive control for all five sets of primers.

### Flow cytometric analysis of protein expression

For analysis of VEGFR-1, -2 and -3, and PDGFR-β protein expression in leiomyosarcoma, 1×10^5^ cells were seeded in 100 mm plates and cultured in DMEM/0.1% FCS for 12 h. Cells were then washed twice with phosphate buffered saline (PBS) and removed from the plate with HEPES/EDTA buffer after 20 min incubation at 37°C. Cells were washed in PBS/3% BSA and fixed in 1% paraformaldehyde (Sigma-Aldrich Chemie GmbH, Taufkirchen, Germany) for 20 min at room temperature. After washing with PBS/3% BSA and permeabilization buffer (PBS/0.5% saponin/3% BSA) cells were incubated with the following primary antibodies diluted 1:100 in PBS/3% BSA for 30 min at 4°C on a shaking device: mouse monoclonal anti-VEGFR-1 antibody [Flt-1 (C-17), cat. no. sc-316], rabbit polyclonal anti-VEGFR-3 antibody [Flt-4 (C-20), cat. no. sc-321], rabbit anti-PDGFR-β antibody (P-20, cat. no. sc-339; all from Santa Cruz Biotechnology, Inc., Heidelberg, Germany) or mouse monoclonal anti-VEGFR-2 antibody (clone KDR-1, cat. no. V9134; Sigma-Aldrich Chemie GmbH). After two washing steps with permeabilization buffer cells were incubated with secondary phycoerythrin (PE)-coupled goat anti-mouse or anti-rabbit Fab fragment (dilution 1:200 in permeabilization buffer; both from Dianova GmbH, Hamburg, Germany) for 1 h at 4°C. Control cells were stained with mouse IgG1, κ (MOPC-21, cat. no. M5284; Sigma-Aldrich Chemie GmbH) or rabbit (Clone DA1E; Cell Signalling Technology, Inc., Danvers, MA, USA) isotype control antibodies for the primary antibody in combination with the respective PE-Fab fragment. After a final washing step in PBS cells were resuspended in 2 ml PBS and analyzed flow cytometrically. Cells were kept in the dark during preparation.

### Flow cytometric assessment of cell cycle and events with lower than G1 DNA content

The extent of cell death was quantified by staining of the cellular DNA content and determination of the fraction of events with lower DNA content than G1-phase cells (sub G1 fraction). Cells (1×10^6^) were seeded in DMEM/10% FCS and grown for 24 h. Samples were supplied with fresh medium and then pre-exposed to 1 μM PTK787 or DMSO (control) and subsequently treated with VEGF or PDGF for 24, 48, and 72 h. After harvesting with 0.05% Trypsin/0.02% EDTA (PAN-Biotech GmbH, Aidenbach, Germany) cells were washed with PBS and 1×10^6^ cells per sample were fixed with 70% ethanol for 24 h at 4°C. Cells were then washed in PBS and treated with 10 U/ml RNase (Sigma-Aldrich Chemie GmbH) for 20 min at 37°C. Cells were next stained with propidium iodide (PI) (final concentration: 25 μg/ml), and incubated for 15 min. The sub G1 fraction and the fraction of live cells (cells in G1-, S-, and G2/M-phase) were determined flow cytometrically with a flow rate of 300 events/sec.

### Flow cytometric data acquisition and analysis

Flow cytometric measurements were done with a FACScan flow cytometer using CellQuest software (BD Biosciences, San Jose, CA, USA). PE and PI fluorescence were exited with a 488 nm argon laser. Fluorescence emission was measured with a 585/42 nm band pass filter (PE) or a >670 nm long pass filter (PI) and visualized on a logarithmic scale. Data were stored as list mode FCS2.0 files.

### Ligand quantification in cell culture supernatants

The amount of secreted human VEGF-A, PDGF-BB was quantified in cell culture supernatants by specific ELISA kits (R&D Systems, Wiesbaden, Germany). Leiomyosarcoma cells were counted, plated at a 40–50% density and cultured in DMEM with either 10, 1 or 0,1% FCS. After an incubation interval of 24 or 48 h, 1 ml cell culture supernatant was removed and analyzed according to the manufacturer’s protocol. Protein levels are expressed as pg/ml.

### SDS-PAGE and western blotting

Tumor cells were starved for 24 h in DMEM/0.1% FCS and then pre-incubated with 0.1, 1 and 10 μM PTK787 or DMSO to serve as control. Subsequently, cells were stimulated with either VEGF or PDGF for 5, 10, 20, 30, 60 min as described above. Cells were then lysed in Laemmli buffer (Rotiphorese^®^ 10X SDS-PAGE cat. no. 3060.1; Carl Roth GmbH & Co. KG, Karlsruhe, Germany) and denatured for 5 min at 95°C. Protein concentrations were measured with the BCA Protein Assay kit (Thermo Fisher Scientific, Bonn, Germany). Protein (20 μg) of each sample was separated by SDS-PAGE on a 10% polyacrylamide gel in a mini gel chamber (Peqlab Biotechnologie GmbH, Erlangen, Germany). Proteins were transferred onto Protran nitrocellulose membranes (Schleicher & Schuell, Dassel, Germany), probed with an antibody cocktail (PathScan^®^ Multiplex Western Cocktail ; Cell Signaling Technology, Inc.) containing the following antibodies: phospho-p90RSK (Ser380) (9D9) rabbit mAb, phospho-S6 ribosomal protein (Ser235/236) (D57.2.2E) rabbit mAb, phospho-p44/42 MAPK (ERK1/2) (Thr202/Tyr204) (D13.14.4W) XP^®^ rabbit mAb, phospho-AKT (Ser473) (D9E) XP^®^ rabbit mAb as well as eIF4E as protein loading control. Blots were washed, incubated with horseradish peroxidase (HRP)-conjugated secondary antibody (anti-rabbit IgG HRP-linked antibody, dilution 1:2,000; Cell Signaling Technology, Inc.) for 2 h and washed again. ECL-chemiluminescence substrate (ECL Plus Western Blotting Detection System; GE Healthcare GmbH, Freiburg, Germany) was used for detection. Membranes were stripped with Restore Western Blot Stripping Buffer (Pierce/Thermo Fisher Scientific) and re-analysed with either phospho-p38 (Thr180/Tyr182) (Clone D3F9) or rabbit mAb, phospho-FAK (Tyr925) or phospho-paxillin (Tyr118) antibody (dilution 1:1,000; all from Cell Signaling Technology, Inc.) using the same protocol as described above.

### MTT assay

To evaluate the effect of PTK787 on cell growth, leiomyosarcoma cells were seeded into 96-well plates (1×10^3^ cells/well) on day 0 in DMEM/10% FCS. On day 1 medium was changed and cells were exposed to 0.1, 1 or 10 μM PTK787. After incubation for 24, 48 or 72 h at 37°C cell growth was assessed using 3-(4,5-dimethylthiazol-2-yl)-2,5-diphenyltetrazolium bromide (MTT) (Sigma-Aldrich Chemie GmbH) at a final concentration of 0.2 mg/ml. After an incubation of 2 h, medium was removed and cells were dissolved in acidic isopropanol (90% isopropanol, 0.5% sodium dodecyl sulphate, 40 mM HCl). The absorbance of the coloured solution was quantified in a spectrophotometer at 490 nm with isopropanol as reference.

### Tumor cell migration assay

To determine the inhibitory effect of 0.1, 1 and 10 μM PTK787 on leiomyosarcoma cell motility *in vitro*, migration assays were performed using a modified Boyden chamber as previously described (31). Briefly, 1×10^5^ cells were suspended in DMEM/1% FCS and seeded into inserts with 8 μm filter pores (BD Biosciences, Heidelberg, Germany). As chemoattractant 50 ng/ml VEGF-A or 10 ng/ml PDGF-BB diluted in DMEM/10% FCS were used. Control samples were incubated in medium without growth factor addition. After 48 h cells were fixed, migrated cells were stained (Diff-Quick reagent; Dade Behring, Inc., Newark, DE, USA), counted under microscope in four random fields and average cell numbers were calculated.

### Immunohistochemistry of patient samples

Leiomyosarcoma tissue samples were culled from the tissue archives of the Institute of Pathology and Neuropathology, University Mainz, Germany. Immediately after surgery, tissue samples were fixed in buffered formaldehyde (4%; SG Planung, Holzkirchen, Germany) and embedded in paraffin (Sigma-Aldrich Chemie GmbH). The specimens were diagnosed by at least two experienced pathologists as leiomyosarcomas and graded after the FNCLCC grading scheme.

For immunohistochemical staining 5 μm sections were prepared. Stains were performed using the ready-to-use, peroxidase-based EnVision^®^ kit (Dako, Hamburg, Germany) according to the manufacturer’s protocol and developed with the Avidin-Biotin Complex (ABC) method with 3-amino-9-ethylcarbazole (AEC) or 3,3′-diaminobenzidine (DAB) staining solution, respectively. The antibodies used are listed in Table II. The sections were counterstained with haematoxylin and mounted with Aquatex^®^ (Merck, Darmstadt, Germany). In control sections, the primary antibody was either omitted or substituted with non-specific rabbit or mouse immunoglobulins. The specimens were analysed by light microscopy (Zeiss Axiophot; Carl Zeiss Microscopy GmbH, Göttingen, Germany).

## Results

### Expression of VEGFRs and PDGFR-β in leiomyosarcoma tissue specimens

**Figure 1:**
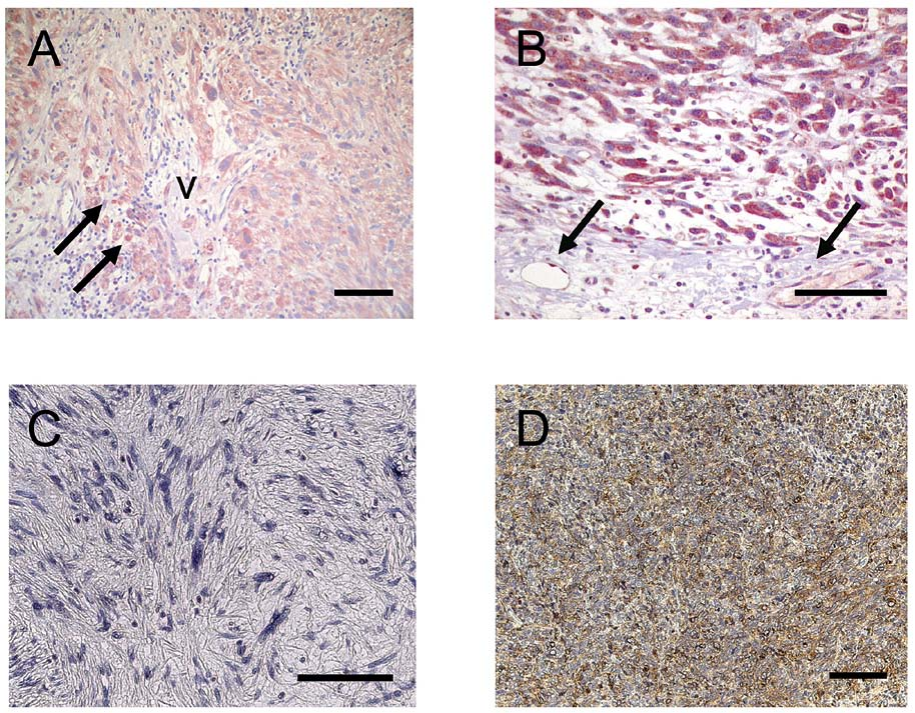
Immunohistochemical investigation of VEGFR-1 (A) shows a weak cytoplasmic expression in pleomorphic sarcoma cells, endothelial cells (v) and numerous macrophages (→) (scale bar, 50 μm). VEGFR-2 (B) is strongly expressed in tumor cells as well as endothelial cells (→) (scale bar, 50 μm). In contrast, VEGFR-3 (C) is completely negative in tumor cells and tumor-associated vessels (scale bar, 50 μm). In addition, PDGFR-β (D) is prominently present in sarcoma cells, and also in perivascular cells so-called pericytes (scale bar, 50 μm).

Immunohistochemical investigations showed a strong expression for VEGFR-1/2 in the cytoplasm as well as the cell membrane indicating a prominent protein expression in tumor cells (Fig. 1A and B). Also vascular endothelial cells expressed VEGFR-2 (Fig. 1B) and to a lesser extent VEGFR-1 (Fig. 1A). In addition, VEGFR-1 was present in tumor-associated macrophages (TAM) (Fig. 1A). On the other hand VEGFR-3 was not present in sarcoma cells and could not be detected in lymphatic vessels in our series (Fig. 1C). In contrast to VEGFR-3, PDGFR-β was prominently expressed in the cytoplasm as well as in the cell membrane of sarcoma cells (Fig. 1D) emphasizing that VEGFR/PDGFR family members play an important role in sarcoma cells. Also PDGFR-β was expressed in perivascular cells as reported previously (19,21,22).

### Expression of VEGFR family members, PDGFR-β and corresponding ligands in leiomyosarcoma cell lines

**Figure 2:**
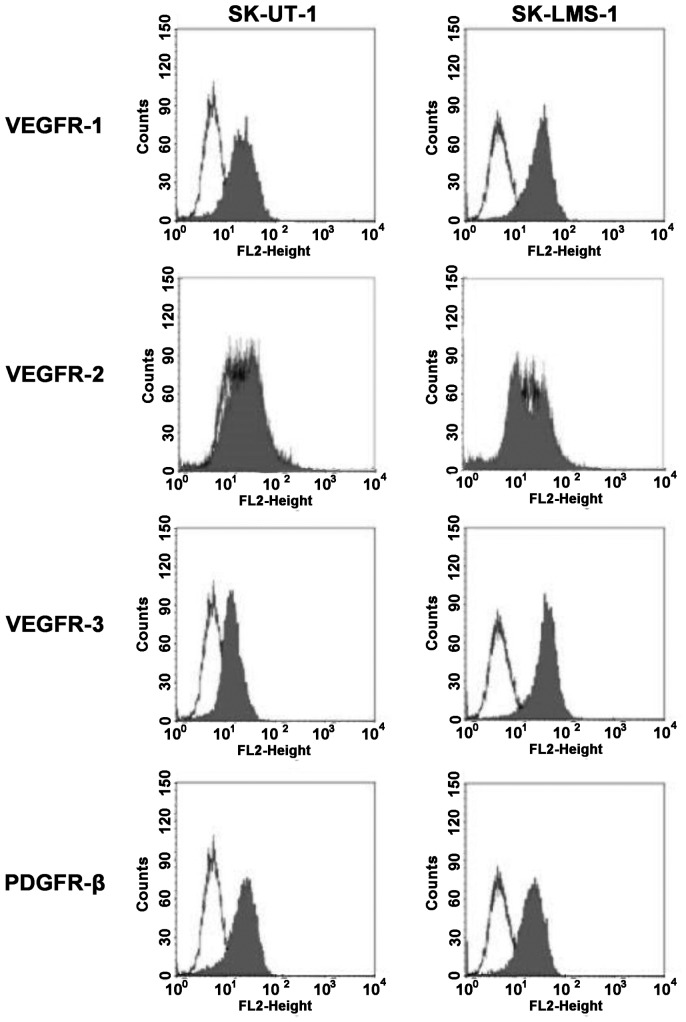
SK-UT-1 and SK-LMS-1 cells express VEGF receptor (VEGFR)-1, -3 and platelet-derived growth factor receptor (PDGFR)-β receptors. VEGFR-2 expression was positive in SK-UT-1, but ambiguous in SK-LMS-1. Line, isotype control staining; filled, antigen staining.

Positive expression of VEGFR family members and PDGFR-β in leiomyosarcoma tissue specimen (Fig. 1) suggested further functional studies on their potential as therapeutic targets for specific tyrosine kinase inhibition. Since it is known that the small molecule inhibitor PTK787 is able to sufficiently block several RTKs (20) we first examined the expression of VEGFR-1, -2 and -3, and PDGFR-β in the two leiomyosarcoma cell lines SK-LMS-1 and SK-UT-1. Analysis of PCR products revealed that VEGFR-1, -2 and -3 were detectable in both sarcoma cell lines as well as in control HUVEC and HL-60 cells. In addition, PDGFR-β mRNA was strongly expressed in both sarcoma cell lines (data not shown). Since the detection of mRNA does not necessarily predict the functional expression of the receptors, we further assessed the protein expression at the cellular surface of the tumor cell lines by flow cytometry (Fig. 2). We were able to show that VEGFR-1 and -3 are strongly expressed in both leiomyosarcoma cell lines whereas VEGFR-2 staining was slightly positive in SK-UT-1 but ambiguous in SK-LMS-1. In addition, PDGFR-β was detectable at the cellular surface of both cell lines.

### Expression of VEGF and PDGF receptor ligands in leiomyosarcoma cell lines

Binding of the corresponding ligands causes receptor activation and subsequent intracellular signalling. Therefore, expression and secretion of corresponding growth factors for the RTKs are essential for their functional activity. Thus, we investigated the secretion of VEGF-A and PDGF-BB for both leiomyosarcoma cell lines with specific ELISA assays. We could detect high amounts of VEGF-A in SK-LMS-1 (1278±148.4 pg/ml after 24 h) which exceeded the detection limit after 48 h. VEGF-A secretion in SK-UT-1 cells was 10.9-fold lower (117±6.9 pg/ml) compared to SK-LMS-1 after 24 h and increased to 371.1±5.2 pg/ml after 48 h. When assessing secreted PDGF-BB levels both cell lines showed comparable amounts after 24 h (SK-UT-1: 36.97±3.6 pg/ml; SK-LMS-1: 26.35±1.04 pg/ml) and 48 h (SK-UT-1: 38.81±12.4 pg/ml; SK-LMS-1: 28.91±1.5 pg/ml) of cell culture.

### The effect of PTK787 on RTK signalling

**Figure 3:**
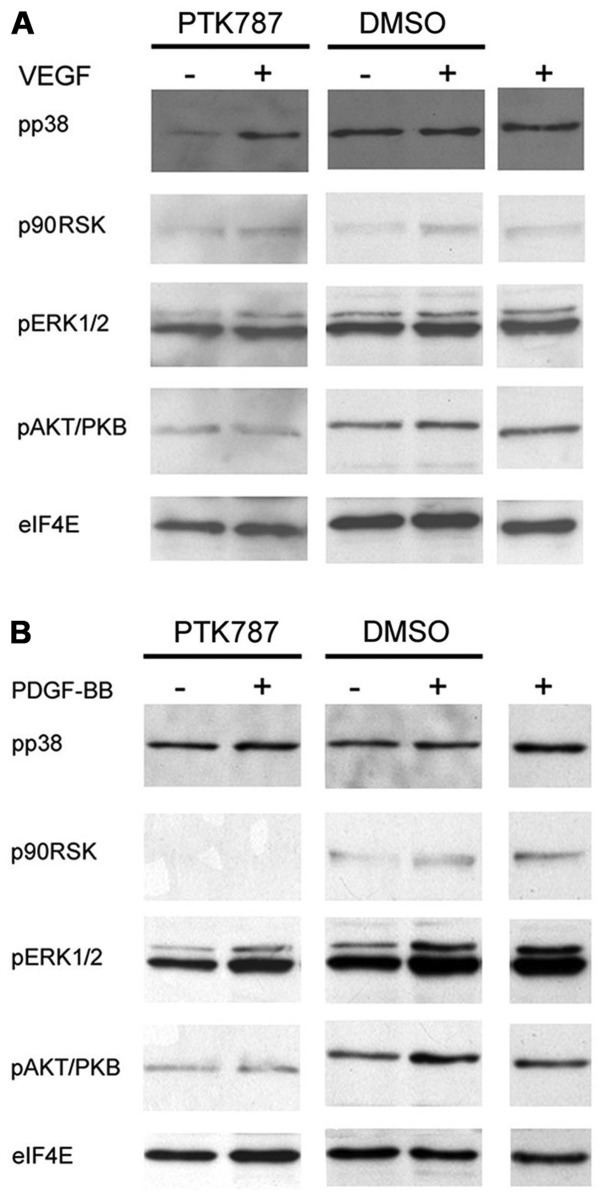
PTK787/ZK222584 (PTK787)-caused effects on signalling cascades (A) in vascular endothelial growth factor (VEGF)-A- and (B) platelet-derived growth factor (PDGF)-BB-treated cells. eIF4E was used as loading control. (A) Activation of signalling cascades is not affected by VEGF-A and/or PTK787 treatment. (B) PTK787 inhibited basal and PDGF-BB-caused ERK1/2, AKT/PKB- and p90RSK-phosphorylation. p, phospho.

We investigated whether PTK787 could interfere with VEGF-A- or PDGF-BB-caused activation of intracellular signalling intermediates, which are known to be involved in VEGFR and PDGFR-signalling and regulate cell proliferation and migration. In Fig. 3 we show representative data gained with SK-UT-1. Results were similar for SK-LMS-1. Cells were incubated with either 0.1, 1 or 10 μM PTK787 or DMSO and subsequently stimulated with growth factors for different time intervals.

VEGF-A stimulation alone or in combination with PTK787 did not affect phosphorylation of ERK1/2 (Fig. 3A). However, both with and without VEGF-A treatment PTK787 seemed to slightly reduce the level of AKT/PKB phosphorylation compared to DMSO control samples. In addition, p38 activation seemed to be reduced upon PTK787 treatment but was brought back to basal levels upon concomitant VEGF-A treatment. Comparable results were obtained for 1 and 10 μM PTK787 and different incubation intervals with growth factors (data not shown). PDGF-BB stimulation (Fig. 3B) increased the phosphorylation of AKT/PKB and ERK1/2 in DMSO control samples. Additional PTK787 treatment reduced the level of ERK1/2 phosphorylation in the presence and absence of PDGF-BB, but a PDGF-BB-caused increase in ERK1/2 phosphorylation remained stable. In line, compared to the DMSO control samples AKT/PKB phosphorylation levels were reduced both for PTK787 treatment alone and for combination treatment of PTK787 and PDGF-BB. Strikingly, PTK787 seemed to completely compensate the stimulatory potential of PDGF-BB on AKT/PKB phosphorylation. Furthermore, p90RSK phosphorylation was abrogated by PTK787 treatment independent of PDGF-BB stimulation.

However, concerning the phosphorylation of p38 no difference could be seen between PTK787 treatment and DMSO alone. Since p38 is a key regulator of cellular migration we also investigated whether alternative signalling pathways for regulation of migration are activated in these cell lines. However, no suppression of either FAK or paxillin phosphorylation, two key regulators of tumor cell migration (32,33), could be observed for both leiomyosarcoma cell lines (data not shown).

### The effect of PTK787 on growth and migration in leiomyosarcoma cell lines

**Figure 4:**
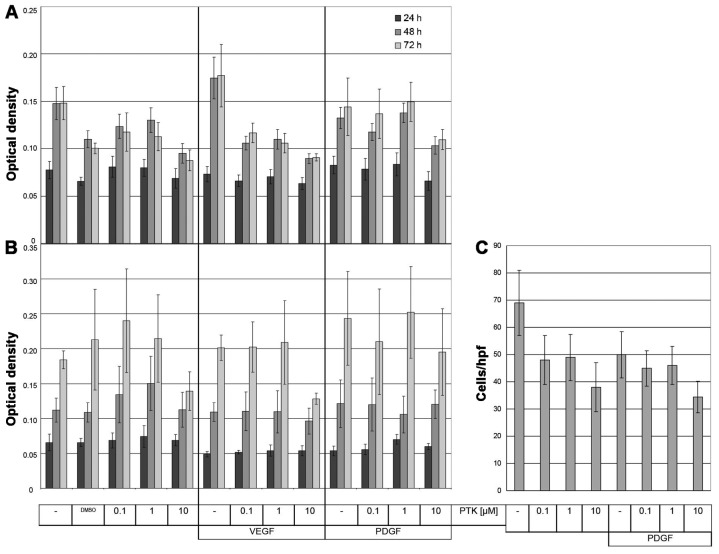
Cell viability of (A) PTK787/ZK222584 (PTK787) (PTK)-pre-treated SK-UT-1 and (B) SK-LMS-1 cells is not changed upon subsequent vascular endothelial growth factor (VEGF)-A- or platelet-derived growth factor (PDGF)-BB-stimulation. (C) PTK787-reduced migration of SK-UT-1 cells measured in a modified Boyden chamber assay after PDGF-BB treatment.

MTT assay was used to assess the cellular growth. Different concentrations of PTK787 were applied and cells were subsequently stimulated with VEGF-A or PDGF-BB. Even with 10 μM PTK787 treatment SK-UT-1 and SK-LMS-1 cells did not show a significant decrease in optical density (OD) compared to control samples (Fig. 4A and B). A positive effect on SK-UT-1 cell growth by VEGF-A treatment was completely compensated by 10 μM PTK787 treatment reaching OD levels similar to samples without VEGF-A addition (Fig. 4A). In SK-LMS-1 VEGF-A treatment had no effect on cell growth. The presence of PDGF-BB alone or in combination with PTK787 treatment caused no difference in OD compared to the respective control samples (Fig. 4A and B).

The modified Boyden chamber assay was applied to assess the effect of PTK787 on cell migration. In Fig. 4C a representative example is shown. Although PTK787 treatment reduced SK-UT-1 cell migration when applied alone, we could not observe a further effect upon PDGF-BB (Fig. 4C) or VEGF-A stimulation (data not shown). Similar results were obtained for SK-LMS-1 (data not shown). Therefore, VEGF-A or PDGF-BB treatment does not affect PTK787-reduced migration of SK-UT-1 and SK-LMS-1 leiomyosarcoma cell lines.

### Increase in sub G1 fraction upon PTK787/growth factor treatment

**Figure 5:**
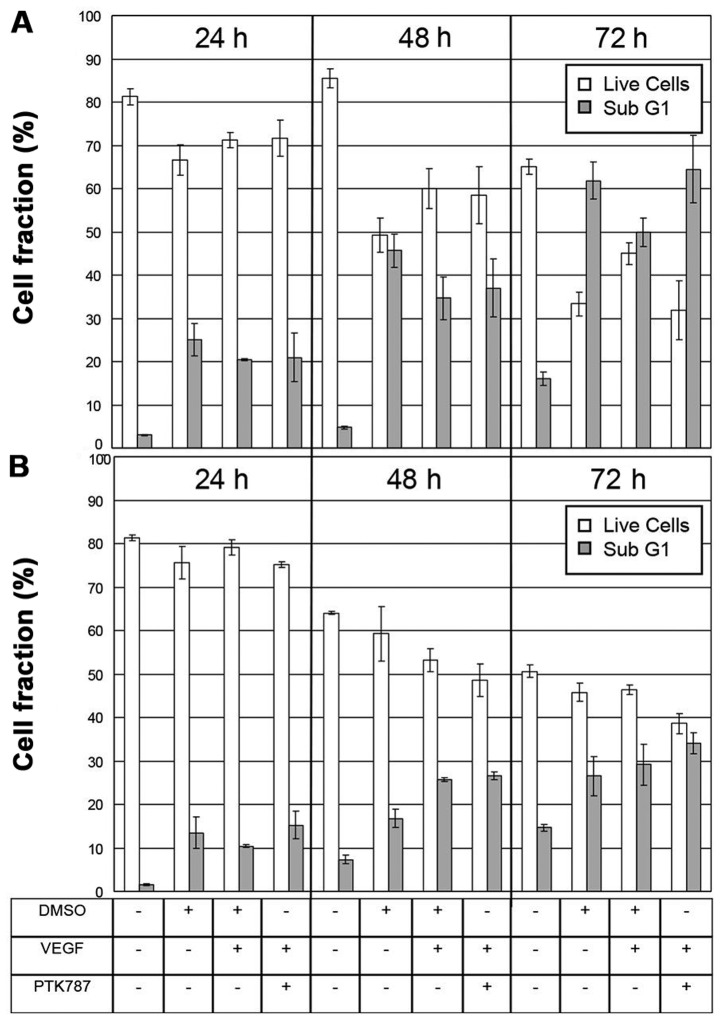
Vascular endothelial growth factor (VEGF)-A-treated (A) SK-UT-1 and (B) SK-LMS-1 cells show apoptotic cell death upon PTK787/ZK222584 (PTK787) treatment. The live cell fraction was determined by gating the cells in G1-, S- and G2/M phase.

Compared to VEGF-A stimulated cells additional PTK787 treatment increased the sub G1 fraction from 49.9±3.3% to 64.5±7.8% in SK-UT-1 (Fig. 5A) and from 29.2±4.8% to 34.1±2.4% in SK-LMS-1 cells (Fig. 5B) after 72 h of incubation. The fraction of live cells (defined as the sum of G1-, S- and G2/M-cell fractions) decreased in SK-UT-1 from 45±2.5% to 31.9±6.9% and in SK-LMS-1 from 46.4±1% to 38.6±2.4%. However, both cell lines were insensitive to PDGF-BB treatment alone and in combination with PTK787 (data not shown). The 48 and 72 h VEGF-A treatment seemed to increase the fraction of live cells and decrease the number of events with lower than G1 cell content only in SK-UT-1 (Fig. 5A) but not in SK-LMS-1 (Fig. 5B).

## Discussion

Conventional therapy of leiomyosarcomas is of limited effect (3). Therefore it is essential to identify new potential therapeutic approaches to improve patient outcome. Targeting the tumor and its vasculature by specific anti-angiogenic drugs has emerged as promising tool to disrupt the outgrowth of new blood vessels, and subsequently the nutrient supply of tumor cells and to directly inhibit tumor growth (4). However, in anti-angiogenic therapy biomarkers to select responders are not available (34).

In the present study, we have demonstrated expression of key proteins for angiogenesis in leiomyosarcoma cells. Strong expression of VEGFR-1, -2 and PDGFR-β in tumor and endothelial cells (Fig. 1) may thus represent the prerequisite for response to inhibition with the multi-targeting anti-angiogenic small molecule inhibitor PTK787. Furthermore, prominent expression of PDGFR-β in perivascular cells/pericytes (Fig. 1D) may represent a complimentary target for efficacious anti-angiogenic therapy by causing pericyte detachment, resulting in immature vessels that are prone to regression (19). The availability of PTK787 target proteins in patient tissue led us to investigate the role and function of PTK787 in a leiomyosarcoma cell culture model to outline the potential of PTK787 for therapy of leiomyosarcoma patients.

We confirmed concomitant expression of angiogenic receptors (VEGFR-1, -2, -3, PDGFR-β, data not shown) and the corresponding ligands (VEGF-A, PDGF-BB) in leiomyosarcoma cell lines SK-UT-1 and SK-LMS-1. In other tumor cell lines it was previously shown that the VEGF/VEGFR system represents an autocrine stimulatory unit (7). Therefore, we investigated the cellular effects of inhibition of VEGFRs with PTK787 upon stimulation with VEGF-A and PDGF-BB.

Our data indicate that upon VEGFR stimulation with VEGF-A the growth-inhibitory effects of PTK787 are predominantly achieved through induction of cell death. This observation is in agreement with other studies that showed an increase in apoptotic cell death upon PTK787 treatment in chronic lymphocytic leukemia (5) and upon PTK787 addition to IFN/5-FU therapy or hypoxia in hepatocellular carcinoma cell lines (35,36).

PTK787 does not display absolute selectivity for the VEGFRs but also blocks the activity of, e.g., PDGFR-β at higher concentrations (20). Despite prominent expression of PDGFRs in SK-UT-1 and SK-LMS-1 (data not shown) cell death was not affected by PTK787 treatment in PDGF-BB activated cells (Fig. 5). However, this finding was accompanied by PDGF-BB-caused phosphorylation of AKT/PKB (cell survival pathway) and ERK1/2 (cell proliferation pathway) and a reversion of these phosphorylation events by PTK787 treatment (Fig. 3). Therefore, we provide evidence that the antitumor efficiency of PTK787 may not only be mediated by AKT-related pathways regulating cell survival (36) but also by affecting cell proliferation (35) via ERK1/2 signalling. In addition, our study emphasizes that PTK787 effectively counteracts PDGF-BB-induced signalling in tumor cells despite a relatively low inhibitory effect for PDGFRs [IC_50_=580 nM vs. VEGFR-1 IC_50_, 77 nM; VEGFR-2 IC_50_, 37 nM (24)]. Therefore, the expression of PDGFRs in leiomyosarcoma cells is likely to significantly participate in tumorigenesis.

However, the lack of induction of cell death upon PDGF-BB/PTK787 treatment raises the intriguing possibilities: I) that the level of PTK787-caused inhibition of cell signalling is not sufficient to result in a significant cellular response; and/or II) that further PDGF-BB-activated signalling cascades are involved in compensation pathways. Such a compensation mechanism or switch may significantly contribute to therapy resistance, which might be counteracted by a combination of anti-angiogenic drugs with conventional or further target-specific treatment (20,35,37).

The lack of VEGF-A-caused effects on phosphorylation of signalling proteins (Fig. 3A) may at least in part be due to a high level of VEGF-A secretion particularly in SK-LMS-1 cells, which probably results in autocrine activation of VEGFR kinase activity and thereby interferes with an effective exogenous supplementation with VEGF-A. Only in VEGF-A low expressing SK-UT-1 cells, VEGF-A treatment resulted in an increase in cell growth (Fig. 4A) and in the number of live cells and in decreased cell death (Fig. 5A). Similarly, a mitogenic response to exogenous VEGF has been shown in different tumor entities, e.g., pancreatic carcinoma, chorioncarcinoma and melanoma (11–13).

The VEGF-A-caused increase in cell growth was reversed by PTK787 to basal levels (Fig. 4A). Furthermore, other studies showed that exogenous VEGF-A compensates a reduction in cell growth caused by VEGFR inhibition with neutralizing antibodies or VEGF ablation with oligonucleotides (7). However, PTK787 treatment of VEGF-A-stimulated SK-UT-1 and SK-LMS-1 did not affect signalling proteins studied herein. Our observations contrast in part with those of previous publications, where PTK787 inhibited VEGF-induced ERK-phosphorylation and cell proliferation of multiple myeloma cell lines (6) and in Chinese hamster ovary cells (25). Other studies showed in hepatocellular carcinoma cell lines that PTK787 treatment alone reduced AKT-phosphorylation, Cyclin D1 and anti-apoptotic Bcl-2 protein expression, which correlated with cell cycle retardation/arrest and reduced cell growth (36). Further studies have to reveal the signalling pathways involved in reverting the VEGF-A-caused increase in cell growth by PTK787 in leiomyosarcoma cell lines. Similarly, the compensating mechanisms that prevent PDGF-BB-treated cells from PTK787-caused cell death despite an efficient inhibition of key proteins in survival pathways (AKT/PKB and p90RSK) have to be further investigated.

Signalling pathways responsible for cell migration were impaired by PTK787 (p38) but not in combination with growth factors (p38 and FAK/paxillin), which correlated with the lack of PTK787 activity on cell migration of VEGF-A- or PDGF-BB-treated cells in Boyden chamber assays. However, in multiple myeloma cell lines PTK787 blocks VEGF-caused cell migration at a concentration of 1 μM (6). In hepatocellular carcinoma cell lines PTK787 treatment reduced expression of migration-related proteins Rac1 and Rho and significantly inhibited cell migration at higher concentrations than studied herein (>20 μM). In addition, cell migration of human leukemic cells was inhibited by anti-VEGFR-1 antibody and VEGFR-2 neutralizing antibody IMC-1C11 suggesting that both VEGFR-1 and -2 take part in regulation of migration (8). However, other authors provide evidence that mainly VEGFR-1 is responsible for regulation of cell migration (38) whereas VEGFR-2 mediates mitogenic signalling, growth and survival. In the present study we found prominent expression of VEGFR-1/-2 in SK-UT-1 and of VEGFR-1 in SK-LMS-1 cell lines, which seemed to be sufficient for inhibition of cell migration by PTK787 (Fig. 4C). However, activation of cell signalling via the VEGFR/VEGF-A or PDGFR-β/PDGF-BB system as well as concomitant PTK787-treatement was shown to be insufficient in effectively reduce migration of leiomyosarcoma cell lines.

In summary, we have shown that both leiomyosarcoma cell lines and patient leiomyosarcoma specimens express members of the VEGFR and PDGFR tyrosine kinase family and their cognate ligands VEGF-A and PDGF-BB that are the key players in angiogenesis for providing tumor nutrient supply. The VEGFR low-molecular weight inhibitor PTK787 has limited impact on leiomyosarcoma cell lines in terms of inhibition of signalling pathways responsible for cell proliferation and cell survival resulting in an induction of cell death. These observations support the notion that anti-angiogenic therapy with PTK787 may be a new therapeutic option for leiomyosarcoma patients with positive expression of PTK787 target molecules. However, compared to blocking angiogenesis by other anti-angiogenic drugs, e.g., bevacizumab, the addition of PTK787 to chemotherapy was less effective in clinical trials (14). On the other hand, two recent phase III clinical studies suggested that a high serum lactate dehydrogenase (LH) level might be useful as a predictive marker for response to PTK787 treatment (18,28). Further *in vitro*, *in vivo* and clinical studies are needed to reveal the involvement of PTK787 target proteins and potential predictive markers for response to treatment. The expression level and interplay of angiogenic growth factor receptors and their cognate ligands in tumor cells, the surrounding endothelial cells and perivascular cells/pericytes have to be taken into consideration offering new strategies to overcome drug resistance by target-specific anticancer therapy.

## Figures and Tables

**Table I:** Gene specific primers for RT-PCR.

Gene name	Sequence (5′→3′)	Size (bp)
*VEGFR-1*	F: ATT TGT GAT TTT GGC CTT GC R: CAG GCT CAT GAA CTT GAA AGC	555
*VEGFR-2*	F: GTG ACC AAC ATG GAG TCG TG R: CCA GAG ATT CCA TGC CAC TT	630
*VEGFR-3*	F: TCC TTG TCG GTA CCG GCG TC R: GAG GAT CTT GAG CTC CGA CA	368
*PDGFR-β*	F: TGA CCA CCC AGC CAT CCT TC R: GAG GAG GTG TTG ACT TCA TTC	228
*GAPDH*	F: GCG GGG CTC TCC AGA ACA TCA T R: CCA GCC CCA GCG TCA AAG GTG	301

F, forward; R, reverse.

**Table II:** Antibody types and source used in this study.

Antibody	Antigen	Provider	Dilution	Epitope retrieval	Incubation	Control
Mouse IgG1	VEGFR-1 Clone C-17	Santa Cruz Biotechnology, Inc., Heidelberg, Germany	1:100	6×5 min in CB, pH 6.0 at 500 W	Overnight RT	Umbilical vein
Mouse IgG1	VEGFR-2 Clone A-3	Santa Cruz Biotechnology, Inc., Heidelberg, Germany	1:50	40 min in CB, pH 6.0 at 240 W	Overnight RT	Colon carcinoma
Rabbit polyclonal IgG	VEGFR-3 Clone C-20	Santa Cruz Biotechnology, Inc., Heidelberg, Germany	1:50	6×5 min in CB, pH 6.0 at 500 W	Overnight RT	Colon carcinoma Umbilical vein
Rabbit monoclonal IgG	PDGFR-β Clone 28E1	Cell Signaling Technology, Inc., Frankfurt, Germany	1:50	6×5 min in CB, pH 6.0 at 500 W	1 h at RT	Ovarian carcinoma

CB, citrate buffer; RT, room temperature.
